# Supplementary material for: A parasite DNA binding protein with potential to influence disease susceptibility acts as an analogue of mammalian HMGA transcription factors
Source: PLoS One. 2023 Jun 5;18(6):e0286526. doi: 10.1371/journal.pone.0286526 (PMC10241358; doi:10.1371/journal.pone.0286526)
Supplement: S1 Fig — The significantly differentially expressed genes were represented in red with adjusted P value < 0.05 determined by DESeq2. The x-axis represents log2change and the y-axis shows–log10 (adjusted p-values). (DOCX) [file pone.0286526.s001.docx]

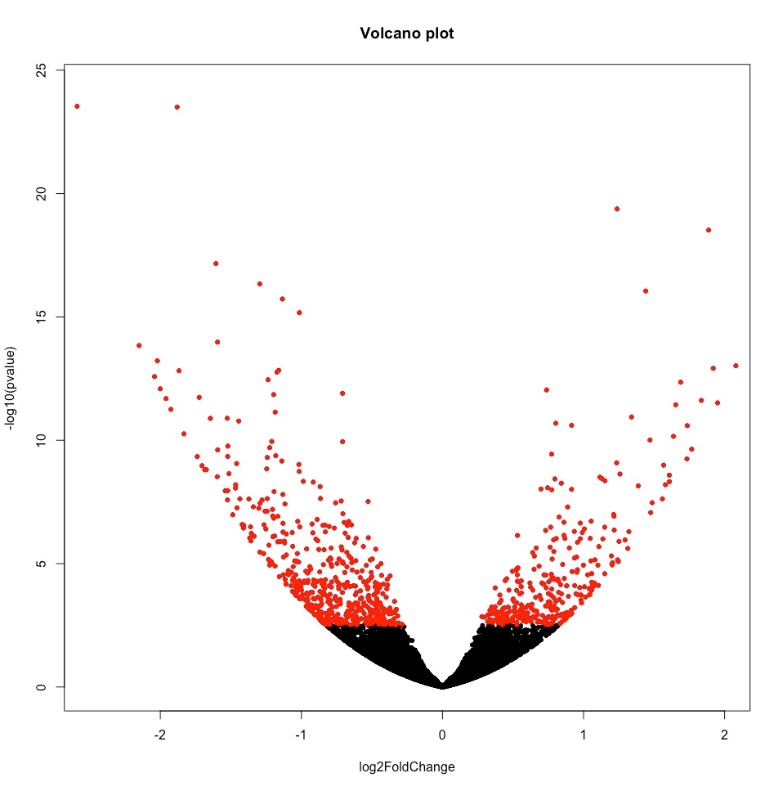


**S2 Figure**: **Volcano plot of differentially expressed genes between control and experimental groups.** Significantly differentially expressed genes are represented in red with adjusted P value < 0.05 determined by DESeq2. The x-axis represents log_2_change and the y-axis shows –log_10_ (adjusted *P*-values).
