# Supplementary figures and images for: A parasite DNA binding protein with potential to influence disease susceptibility acts as an analogue of mammalian HMGA transcription factors
Source: PLoS One. 2023 Jun 5;18(6):e0286526. doi: 10.1371/journal.pone.0286526 (PMC10241358; doi:10.1371/journal.pone.0286526)

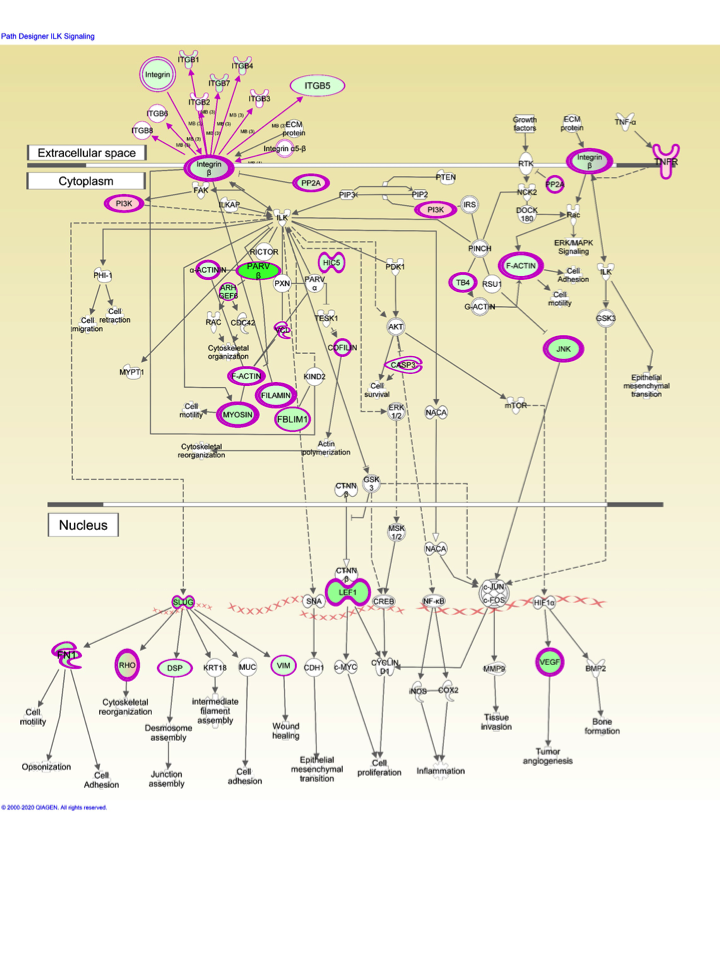

Supplement: S2 Fig — Down-regulated genes are highlighted in green while up-regulated genes are highlighted in red. (TIFF) [file pone.0286526.s002.tiff]
