# Supplementary material for: A parasite DNA binding protein with potential to influence disease susceptibility acts as an analogue of mammalian HMGA transcription factors
Source: PLoS One. 2023 Jun 5;18(6):e0286526. doi: 10.1371/journal.pone.0286526 (PMC10241358; doi:10.1371/journal.pone.0286526)
Supplement: Attachment — Submitted filename: Response to Reviewers.docx [file pone.0286526.s012.docx]

**Response to Reviewers comments**

**Reviewer #1:**

*Abstract*

Introductory part (line 24-34) is too long. It can be cut short and can be discussed in detail in the introduction or discussion sections.it is suggested to use past tense while writing the abstract and result description.

**Response:** The abstract has been shortened and the past tense has now been used to describe results here and throughout the manuscript: see lines 24-41.

lines 39-41... "Validation at the protein level of modulated expression of a gene (GULP1) potentially linked to disease susceptibility was obtained for TashAT2 transfected BoMac, and between an infected Sahiwal and Holstein cell line".... what were the findings???/ rather than describing the methadology, mention the major findings.

**Response:** This sentence has been rewritten to describe the findings: lines 37-39 “Altered protein levels encoded by one of these genes (*GULP1*) was strongly linked to expression of *TashAT2* in BoMac cells and was demonstrated to be higher in infected Holstein leucocytes compared to Sahiwal.”

Introduction is too long. Restrict it to the back ground of study and take explanations to discussion.

**Response:** The introduction has been substantially shortened (by 17 lines), and the explanations made moved/merged with end of discussion: lines 698-699 and lines 709-712 (revised document).

*Methods*
please provide the country along with the suppliers for cell lines and chemicals.
Please provide the web links for the databases used in this study.

**Response:** A reference to the generation of the infected cell lines was added to methods section, and the country of origin of the *T. annulata* isolate used to generate the lines stipulated: see lines 101-103 “The infected bovine cell lines derived from Sahiwal (S3) and Holstein (H3) cattle were generated and cultured as described [28, 43], and represent the Hissar (India) isolate of *T. annulata*.”

Suppliers and country for chemicals/reagents/kits have been added to the methods section, throughout. References, web links and accession numbers have been added to the data availability section: lines 257-263.

*Results*
Table 1 and 2 are cropped and can not be read completely.

**Response:** The issue with Tables was due to their placement in the body text and they were cropped when the journal website built the PDF; the Tables have been reformatted and can be seen fully in the tracked/corrected Word document version of the manuscript. If further modification is required or they need to be loaded up as separate files to be observed, we will be happy to do so.
